# Mitogenome assembly and phylogenetic relationships of *Phalaris arundinacea*

**DOI:** 10.3389/fpls.2026.1926603

**Published:** 2026-08-26

**Authors:** Ze Teng, Chunyu Tian, Yanting Yang, Wenlong Gong, Zhiyong Li, Xiaoming Bai, Zinian Wu

**Affiliations:** 1 Pratacultural College, Gansu Agricultural University, Lanzhou, China; 2 Institute of Grassland Research, Chinese Academy of Agricultural Sciences, Hohhot, China; 3 Key Laboratory of Grassland Resources and Utilization of Ministry of Agriculture, Hohhot, China

**Keywords:** gene transfer, mitogenome, *Phalaris arundinacea*, RNA editing, RSCU

## Abstract

**Introduction:**

As a perennial herb of Poaceae, *Phalaris arundinacea* plays key roles in grazing, production, and soil and water conservation because of its well-developed rhizomes and seed dispersal. We assembled and annotated the first mitogenome of *P. arundinacea* to support evolutionary and taxonomic research.

**Methods:**

We assembled and annotated the first complete mitochondrial genome of *P. arundinacea* by integrating Illumina short reads with Nanopore long reads via a hybrid assembly strategy. The genome architecture was comprehensively characterized, encompassing codon usage bias, repetitive sequence organization, and inter-organellar genetic exchange with the chloroplast genome.

**Results and discussion:**

Assembly of the *P. arundinacea* mitogenome revealed two circular structures with a combined length of 526,717 bp. The genome comprised a set of 37 protein-coding genes (PCGs), 27 tRNAs, and 8 rRNAs, with the rRNA genes exhibiting full assembly (100% coverage). The mitochondrial genome contained 154 forward and 164 palindromic repeats, along with 25 tandem repeats and 124 simple sequence repeats (SSRs). Notably, 102 SSRs were distributed on contig1, predominantly in tetrameric form. Furthermore, 376 RNA editing sites were predicted. A total of 104 fragments were integrated into the mitochondrial genome from the chloroplast, amounting to 55,866 bp of transferred sequence. Finally, phylogenetic analysis of 28 plant mitogenomes placed *P. arundinacea* closest to species within the genus *Poa* (*P. chaixii* and *P. pratensis*). Comparative analysis of non-synonymous-to-synonymous substitution rate (Ka/Ks) ratios across divergent species revealed that the mitochondrial genome of *P. arundinacea* underwent stabilizing evolutionary dynamics, characterized by predominant purifying selection with several lineage-specific variations in selective pressure. Our findings support the close phylogenetic relationship between P. arundinacea and species of the genus Poa and provide a reference mitochondrial genome resource for future comparative studies within Phalaris that incorporate broader taxon sampling. These results support deeper phylogenetic investigations of *P. arundinacea* and facilitate future work on its germplasm characterization and applied use.

## Introduction

As a perennial herb of the Gramineae family, *Phalaris arundinacea* exhibits unique biological characteristics and a wide distribution range. The developed rhizome system, seed dispersal ability, and wide adaptability not only make it useful for grazing, production, and soil and water conservation, but also attract extensive attention as a bioenergy material. Owing to its strong adaptability, *P. arundinacea* thrives on marginal lands unsuitable for conventional agriculture and is naturally distributed across temperate and Neotropical regions (Sahramaa et al., 2004; Perdereau et al., 2017; Xiong et al., 2020). Meanwhile, *P. arundinacea* is regarded as an invasive species in some areas, competing with local crops for resources such as light, fertilizer, and water throughout its growth cycle (Apfelbaum and Sams, 1987; Lavergne et al., 2010). *P. arundinacea* is a polyploid plant with extensive variations in ploidy level, habitat, and geographical distribution. Initial molecular phylogenetic analyses assigned *P. arundinacea* to the Aveneae-Poeae complex within the subfamily Pooideae (Quintanar et al., 2007; Schneider et al., 2009; Saarela et al., 2015). Currently, 20 species of *Phalaris* have been identified (Voshell and Hilu, 2014). This includes both endemic species, such as *P. rotgesii*, *P. truncata*, *P. californica*, and *P. maderensis*, and the widespread cosmopolitan species *P. arundinacea* L (Galatowitsch et al., 1999; Lavergne and Molofsky, 2004; Thomsen et al., 2012).

As key cellular organelles, mitochondria and chloroplasts perform vital functions in plants, ranging from energy conversion and metabolism to developmental signaling (Chen and Butow, 2005). Compared with whole-genome sequencing, organellar genomes offer greater cost-effectiveness and serve as robust foundations for evolutionary studies (Sun et al., 2023). Plant mitochondrial genomes are characterized by considerable structural diversity—existing in both circular and linear forms—and are shaped by frequent repeat-mediated recombination, the acquisition of exogenous DNA, and the deletion of endogenous sequences (Bergthorsson et al., 2003; Sloan, 2013; Wynn and Christensen, 2019). Notably, plant mitochondrial genomes evolve rapidly in structure while slowly in sequence (Palmer and Herbon, 1988), making them particularly suitable for resolving structural rearrangements and inter-organellar DNA transfer events that are often undetectable using chloroplast or nuclear markers alone; Furthermore, plant cells frequently exhibit bidirectional DNA exchange between their chloroplast and mitochondrial genomes (Allen, 2015; Turmel et al., 2016). Collectively, these features make the mitochondrial genome a powerful tool for investigating structural variation, intracellular gene transfer, and phylogenetic relationships within plant groups (Zou et al., 2025).

In *P. arundinacea*, previous genomic research has achieved partial progress. The chloroplast genome has been utilized for species and ploidy comparisons (Xiong et al., 2020), and nuclear genome-based molecular markers such as EST-SSR and ISSR have revealed high genetic diversity within populations. However, these markers have limitations in elucidating the molecular mechanisms underlying complex adaptive traits. Crucially, a significant knowledge gap remains regarding the sequence architecture, structural configuration, and evolutionary trajectory of the *P. arundinacea* mitochondrial genome. Given its complex ecological adaptability and polyploid nature, key genes within the mitochondrial genome may regulate adaptive responses to diverse environments. Moreover, owing to the evident variations in its morphological characteristics and the crucial role that polyploidy plays in its evolutionary process (such as tetraploid and hexaploid *P. arundinacea*), the mitochondrial genome has the potential to provide novel insights into grass species formation, chromosome evolution, and biogeography (Xiong et al., 2020).

To address this gap, we assembled and annotated the first complete mitochondrial genome of P. arundinacea using a hybrid strategy integrating Illumina short reads with Nanopore long reads. Subsequently, with the help of these two platforms, we carried out detailed annotation of the assembled mitochondrial genome. Subsequent analyses encompassed multiple facets of the *P. arundinacea* mitogenomic architecture, including codon usage bias, repetitive sequence organization, and inter-organellar genetic exchange with the chloroplast genome. By constructing and analyzing a phylogenetic tree, we identified crucial clues to uncover the evolutionary history of *P. arundinacea*.

## Materials and methods

### DNA extraction, genome sequencing and assembly

Fresh leaf tissues of *P. arundinacea* were harvested from the National Perennial Forage Germplasm Resource Nursery located in Hohhot, Inner Mongolia, China (40.57°N, 111.93°E). To isolate high-quality genomic DNA, a modified protocol integrating the conventional CTAB method with the Qiagen Blood and Cell Culture DNA Kit (Cat. no. 13323) was implemented, followed by an ethanol precipitation step. Dual-platform sequencing was conducted using the Illumina NovaSeq 6000 and Oxford Nanopore PromethION systems. This combined approach yielded exactly 52,328,441 short reads (15,796,442,260 bases) from the Illumina platform and 1,578,326 long reads (17,189,341,717 bases) via Nanopore sequencing. For mitogenome reconstruction, the long Nanopore reads were initially aligned against a customized plant reference gene database (https://github.com/xul962464/plant_mt_ref_gene) utilizing minimap2 v2.1 (Sahlin, 2023). The resulting draft assembly underwent an error-correction polishing phase using Canu v2.2 (Koren et al., 2017). Subsequently, this polished sequence functioned as a reference framework to align the short Illumina reads using Bowtie2 v2.3.5.1 (Langmead and Salzberg, 2012). A comprehensive hybrid assembly was then constructed with Unicycler v0.4.8 (Wick et al., 2017) operating under standard default configurations. Finally, the structural integrity and circular conformation of the assembled mitogenome were visually validated and manually curated through the Bandage v0.8.1 graphical interface (Wick et al., 2015).

### Genome annotation

For the initial annotation of the mitochondrial genome, we employed the GeSeq web server (Tillich et al., 2017), utilizing existing homologous Poaceae mitogenome records from the NCBI database as reference frameworks. The gene structure was subsequently predicted using PMGA (Li et al., 2025). The annotation results from both GeSeq and PMGA were integrated to generate a comprehensive and refined gene annotation profile, and Geneious (v2024.0.5) (Kearse et al., 2012) was employed for the manual curation and refinement of the initial annotation results. Finally, the genomic map was constructed using the OGDRAW (Organellar Genome Draw) software (v1.3.1) (Greiner et al., 2019).

### Repeat sequence identification

Microsatellites (SSRs) within the *P. arundinacea* mitochondrial genome were mined using the MISA tool (Beier et al., 2017). The detection thresholds were rigorously standardized to a minimum of 10, 5, and 4 repeat units for mono-, di-, and trinucleotides, respectively, whereas a strict minimum of three iterations was required for tetra-, penta-, and hexanucleotide motifs. For tandem repeat identification, the FASTA-formatted assembly was processed using Tandem Repeats Finder (TRF v4.09) (Benson, 1999). The algorithm’s scoring matrix was customized as follows: alignment match = 2, mismatch/indel penalties = 7, match probability (PM) = 80, and indel probability (PI) = 10. Additional constraints included a minimum alignment score of 50 and a maximum period size of 2,000 bp, alongside the activation of the -f (flanking), -d (data), and -m (masked) output parameters. Furthermore, REPuter (Kurtz et al., 2001) was executed to locate four distinct classes of dispersed repeats (forward, palindromic, reverse, and complement). The search constraints were restricted to a minimum length of 12 bp and a maximum Hamming distance of 3. Following stringency filtering, the surviving high-confidence repetitive elements were fully cataloged based on their specific structural classifications, genomic coordinates, and sizes.

### Codon usage bias analysis

The relative synonymous codon usage (RSCU) profiles and amino acid frequencies for the entire set of PCGs were calculated using the CodonW software package (v1.4.4) (Iriarte et al., 2021). Codon usage bias was analyzed using custom Perl scripts, and unique coding sequences (CDS) were selected for subsequent analysis. Subsequently, the results were plotted using R.

### Prediction of RNA editing sites

Potential RNA editing events within the mitochondrial protein-coding genes were identified using unprocessed sequencing data retrieved from SRA database (accession numbers: SRR13516133, SRR13516134, SRR13516135, SRR13516136, SRR13516137, SRR13516138, SRR13516139, SRR13516140, SRR13516141, SRR13516142, SRR13516143, and SRR13516144); https://www.ncbi.nlm.nih.gov/sra/). The alignment of RNA-seq reads to mitochondrial PCGs was executed using BWA v0.7.1 (Li and Durbin, 2010). Subsequently, variant calling was carried out via the SAMtools (v1.17) (Li et al., 2009) and BCFtools (v1.17) (Danecek et al., 2021) pipeline. To identify high-confidence RNA editing events, the resulting SNP profiles were systematically screened and annotated using the REDO toolkit (v1.0) (Wu et al., 2018). To reduce false positives, the DNA sequencing reads were mapped against the *Phalaris arundinacea* mitochondrial genome as the reference. Genomic SNPs were called using BCFtools (v1.17), followed by the exclusion of regions overlapping mRNA editing sites.

### Chloroplast-derived mitochondrial sequence identification

Utilizing the same raw Illumina sequencing library, the plastid genome (cpDNA) was reconstructed through a combined protocol of *de novo* assembly and reference-guided alignment. The sequence assembly was executed using the GetOrganelle (v1.7.7.0) (Jin, 2020) under predefined configurations (-R 50 -k 21,45,65,85,105,115 -P 1000000). For gene prediction, an integrated approach deploying both CPGAVAS2 and PGA was applied (Qu et al., 2019; Shi et al., 2019). The preliminary automated outputs were subsequently merged and subjected to manual refinement within Geneious software to establish the definitive cpDNA annotation. To detect potential intracellular DNA transfers, the assembled chloroplast sequence was compared against the mitogenome via a homology search using BLASTn (v2.16.0) (Chen et al., 2015) with a strict cutoff parameter (E-value ≤ 1e-5). Finally, the spatial distribution and mapping of these plastid-derived fragments across the mitochondrial architecture were graphically represented using TBtools (v2.091) (Chen et al., 2020).

### Selection pressure analysis and nucleotide diversity assessment

Sequence alignments for 27 conserved mitochondrial genes shared among *P. arundinacea* and 25 other Poaceae species were first generated using MAFFT (v7.526) (Katoh and Standley, 2013). These genes included *atp1, atp4, atp6, atp8, atp9, ccmB, ccmC, ccmFC, ccmFN, cox1, cox2, cox3, matR, mttB, nad1, nad2, nad3, nad4, nad4L, nad5, nad7, nad9, rpl16, rps13, rps2, rps3, and sdh4*. Based on the aligned sequences, the nonsynonymous (Ka) and synonymous (Ks) substitution rates were subsequently estimated via KaKs (Calculator v3.0) (Zhang, 2022). Additionally, the nucleotide diversity (Pi) across 30 specific mitochondrial genes in *P. arundinacea*, *Poa pratensis*, and *Poa chaixii* was quantified using DnaSP (v6.12.0) (Rozas et al., 2017).

### Phylogenetic and synteny analysis

To elucidate the phylogenetic relationships, a matrix of 25 shared mitochondrial PCGs (*atp1*, *atp4*, *atp6*, *atp8*, *atp9*, *ccmB*, *ccmC*, *ccmFC*, *ccmFN*, *cox1*, *cox2*, *cox3*, *matR*, *mttB*, *nad1*, *nad2*, *nad3*, *nad4*, *nad4L*, *nad5*, *nad7*, *nad9*, *rpl16*, *rps3*, *sdh4*) was assembled from 26 Poaceae species, with *Arabidopsis thaliana* and *Medicago truncatula* included as outgroup taxa. All reference mitochondrial genome sequences were retrieved from the NCBI repository (detailed in **Supplementary Table 1**). The selected PCGs were concatenated and aligned using MAFFT (v7.131) (Katoh and Standley, 2013). Following model selection via ModelFinder (Kalyaanamoorthy et al., 2017), phylogenetic inference was performed using two distinct approaches. The Maximum Likelihood (ML) tree was generated by RAxML (Stamatakis, 2006) employing the GTRGAMMA model with 1,000 bootstrap replicates, while Bayesian inference (BI) was executed in MrBayes (Huelsenbeck and Ronquist, 2001) under the GTR+F+I+G4 substitution model.

### Synteny analysis

To elucidate genomic collinearity, pairwise sequence alignments were conducted between *P. arundinacea* and three related species (*Poa chaixii*, *Poa pratensis*, and *Triticum aestivum*) utilizing BLASTn (v2.16.0). Subsequently, AliTV (v1.0.6) (Ankenbrand et al., 2017) was employed to detect high-confidence syntenic blocks and construct comparative maps illustrating conserved genomic regions.

## Results

### Genomic features of the *P. arundinacea* mitogenome

**Figure 1:**
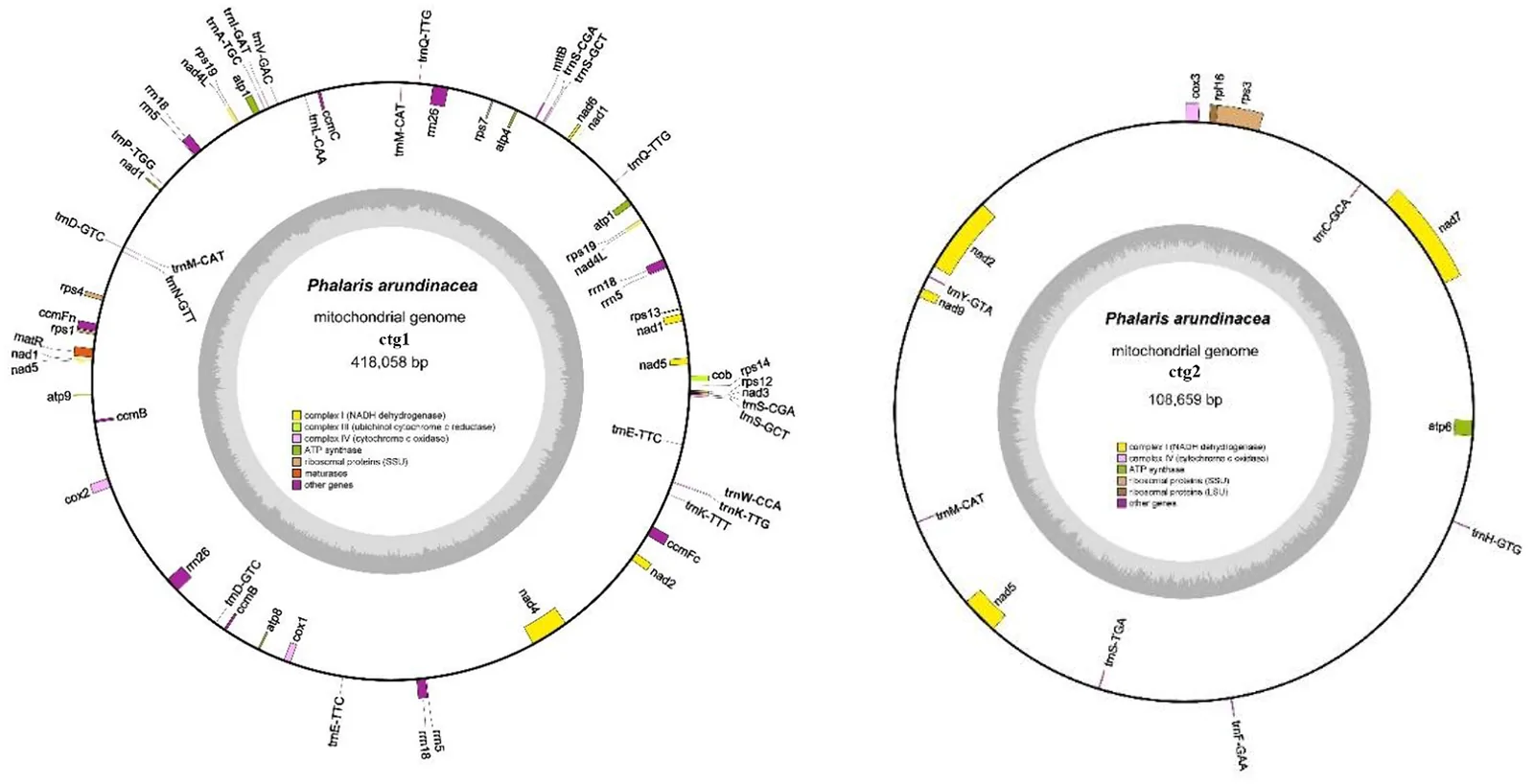
Circular representation of the complete mitochondrial genome of *Phalaris arundinacea*. Depicting a circular map, genes on the outer and inner rings are annotated to indicate forward and reverse transcription directions, respectively. The inner ring features a charcoal grey gradient representing GC content, while various functional gene groups are highlighted with distinct colors.

**Figure 2:**
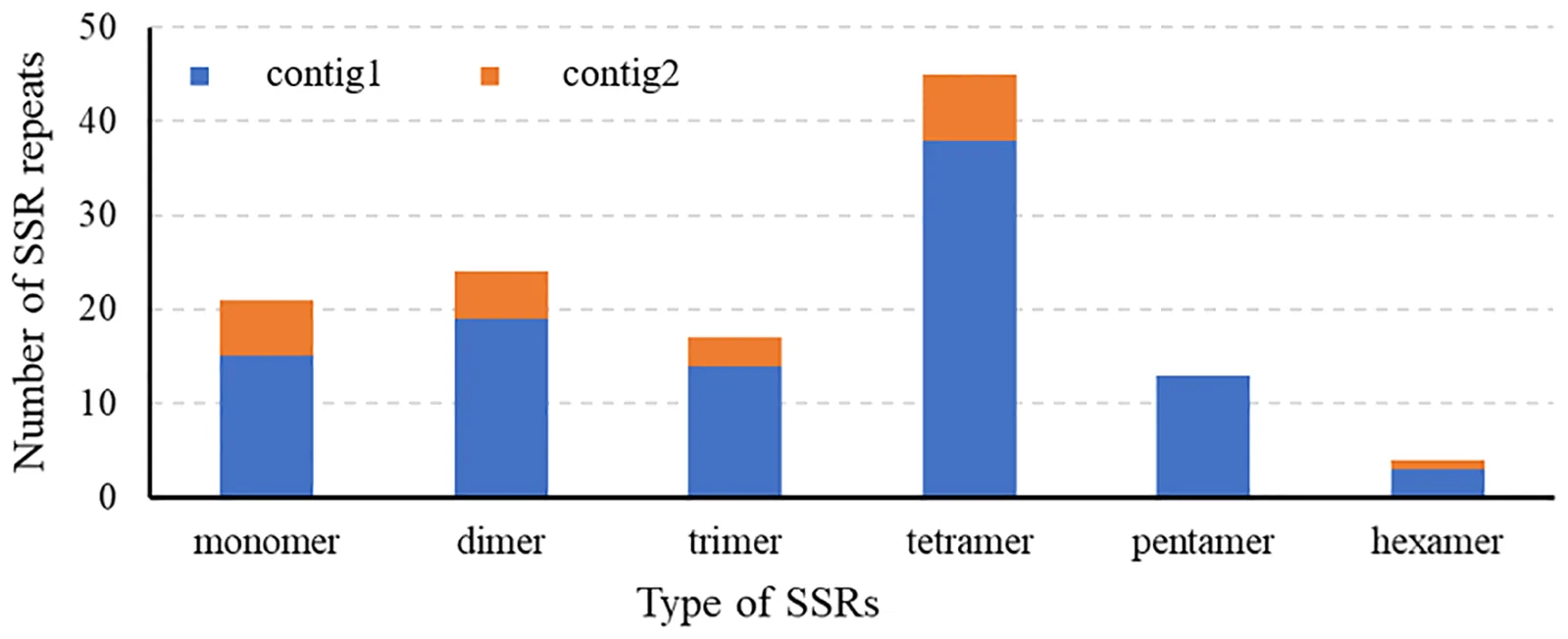
Distribution of SSRs in the *P. arundinacea* mitogenome. The X-axis denotes SSR types, and the Y-axis shows the count of repeat units.

The assembled *P. arundinacea* mitogenome comprises two complex, circular structures, spanning a total length of 526,717 bp with a GC content of 44.46% (**Figures 1**, **2**). Annotation identified a total of 72 functional genes (**Supplementary Tables 2**, **S3**), which are unevenly distributed between contig1(ctg1) (58 genes) and contig2 (ctg2) (14 genes). The coding repertoire consists of 37 unique PCGs, 27 tRNAs, and 8 rRNAs. Among the PCGs, 16 are classified as core genes, including six ATP synthase subunits (with two copies of *atp1*, plus *atp4, atp6, atp8*, and *atp9*), five cytochrome c biogenesis genes (*ccmC, ccmFc, ccmFn*, and duplicated *ccmB*), *cob*, *cox1–3*, and the maturase *matR*. The remaining 21 PCGs are categorized as variable genes, encompassing the transport protein *mttB*, ten NADH dehydrogenase subunits (including duplicated *nad4L* and *nad1-7*, *nad9*), *rpl16*, and notably two copies of *rps19* alongside *rps1, rps3, rps4*, and *rps7, rps12-14*.

The mitochondrial genome assembly of *P. arundinacea* comprised two contigs: ctg1 (418,058 bp, GC content 44.62%) and ctg2 (108,659 bp, GC content 43.85%). ctg1 contained 31 CDS spanning 23,972 bp (GC 42.81%), 21 tRNA genes (1,785 bp, GC 50.14%), and eight rRNA genes (13,242 bp, GC 53.26%). In contrast, ctg2 harbored only six CDS (8,104 bp, GC 42.23%), six tRNA genes (462 bp, GC 53.25%), and no rRNA genes. Notably, the tRNA and rRNA regions exhibited higher GC content than the CDS in both contigs, suggesting distinct evolutionary constraints on non-coding functional elements.

Detailed annotation of gene structure (**Supplementary Table 3**) revealed distinct intron patterns within the *P. arundinacea* mitogenome. Specifically, the genes *nad1, nad2, nad5*, and *nad7* exhibited the most complex structure, each containing four introns, while *nad4* harbored three. *cox2* was found to contain two introns, whereas *ccmFc, rps3, trnA-TGC*, and *trnI-GAT* each possessed a single intron. Gene duplications were predominantly observed among non-coding RNAs. Three copies were identified for *rrn18, rrn5*, and *trnM-CAT*, while two copies were detected for *rrn26* and several tRNA genes (*trnD-GTC, trnE-TTC, trnP-TGG, trnQ-TTG, trnS-CGA*, and *trnS-GCT*). Among PCGs, duplications were restricted to *atp1* and *ccmB*, each present in two copies.

### Characterization of repeat sequences

**Figure 3:**
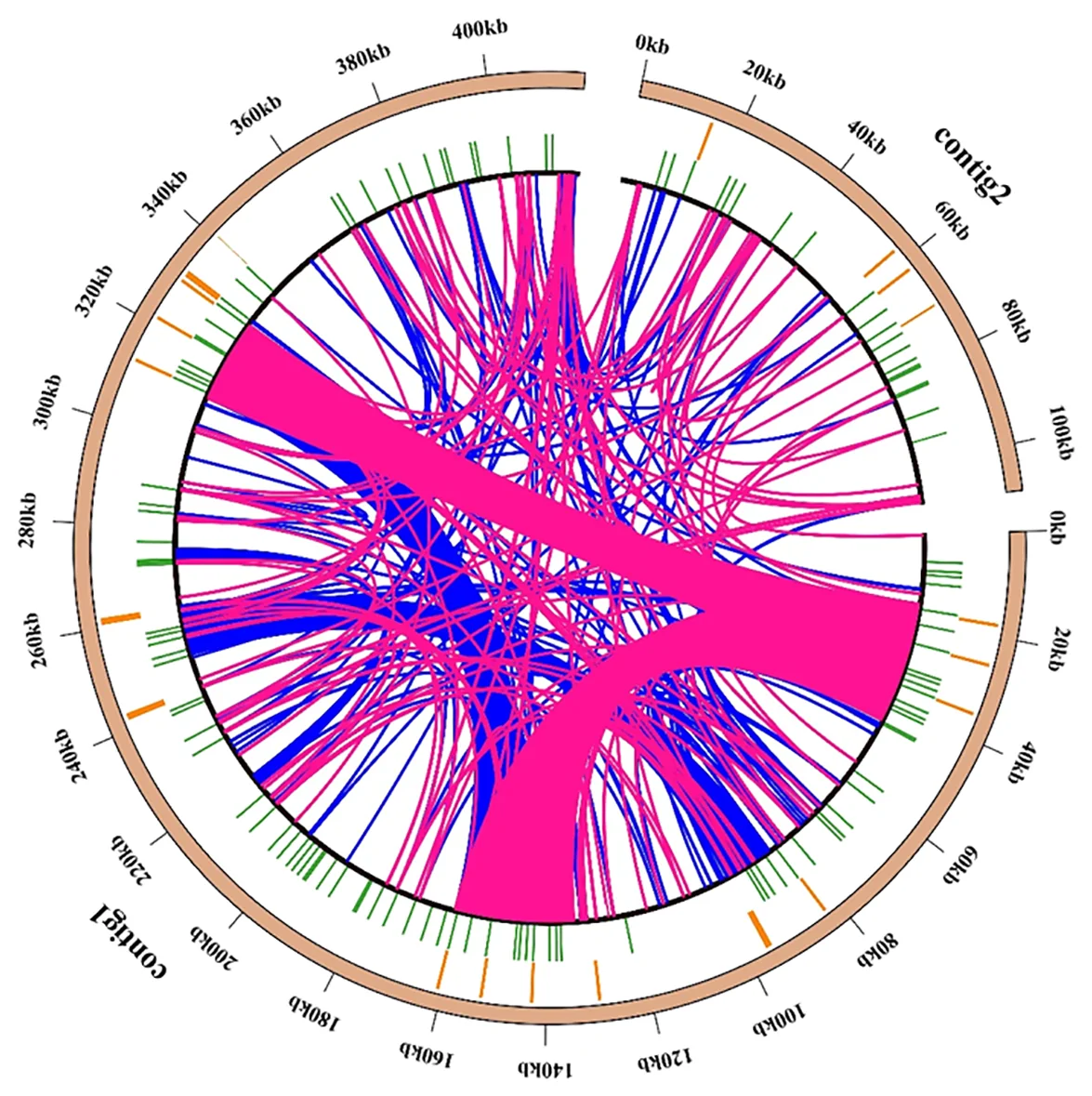
Distribution of dispersed repeats in the *Phalaris arundinacea* mitogenome. From the outermost to the innermost, the concentric circles represent the complete mitochondrial genome sequence, tandem repeats, SSR repeats, and dispersed repeats. Dispersed repeats are illustrated by blue arcs (154 forward repeats) and deep pink arcs (164 palindromic repeats).

**Figure 4:**
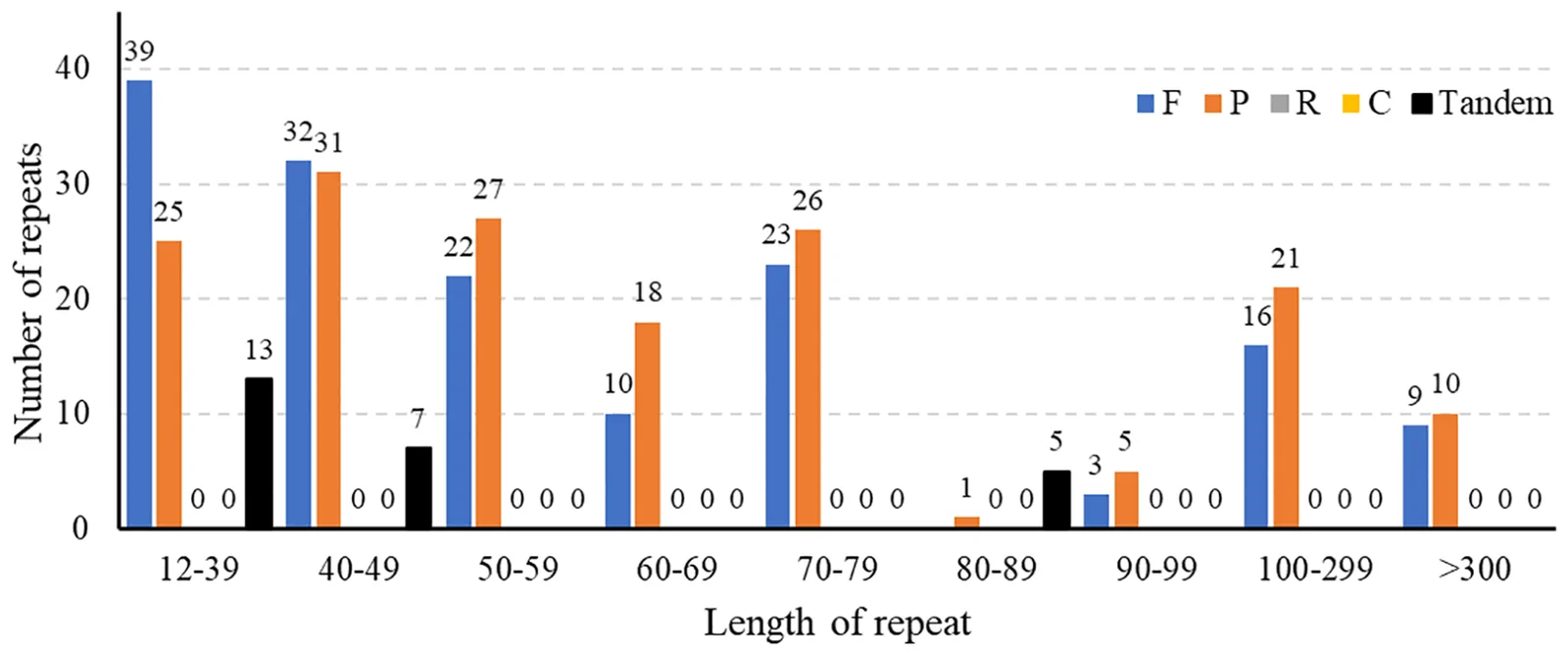
Length distribution of dispersed repeats in the *Phalaris arundinacea* mitogenome. The X-axis categorizes the types of dispersed repeats (F, forward; P, palindromic; R, reverse; C, complement), and the Y-axis shows their corresponding counts.

Repeated sequences are widely distributed in the *P. arundinacea* mitogenome and exist as three types: dispersed repeats, tandem repeats, and SSRs (**Figure 3**). Dispersed repetitive sequences can be classified into four types: forward, palindromic, reverse, and complementary. A total of 318 repetitive sequences (≥28 bp in length) were identified. (**Figure 4**; **Supplementary Table 4**). Among these repeats, 154 pairs (48.40%) were forward repeats, with the remainder (164 pairs) being palindromic repeats. Nineteen dispersed repeat sequences, each exceeding 300 bp in length, were identified. The longest forward repeat sequence located in four genes (two *rrn5* and two *rrn18*) measured 19,468 bp, and the longest palindromic repeat, located in ten genes (two *rrn18*, two *atp1*, two *rrn5*, two *nad4L*, and two *rps19*), measured 27,793 bp. In the shorter intervals (12–39 bp and 40–49 bp), there were relatively large numbers of forward and palindromic repeat sequences. As the length increased, the number decreased. However, in some intervals (such as 70–79 bp and 100–199 bp), the number increased.

Tandem repeats are present in both prokaryotic and eukaryotic genomes. In the *P. arundinacea* mitogenome, we identified 25 tandem repeats ranging from 24 to 153 bp in length. All these tandem repeats resided in intergenic spacer (IGS) regions, with 52% showing perfect sequence identity (100% match). Their copy number variation ranged from 1.9 to 3.2. (**Figure 4**; **Supplementary Table 8**).

A total of 124 SSRs were identified in the *P. arundinacea* mitogenome, distributed on both contigs, of which 102 (82.26%) were found on ctg1 (**Supplementary Table 5**). Tetramers accounted for the largest proportion of 1–6 nucleotide repeat types, representing 36.29%, of which 38 tetramers were present on ctg1 and seven tetramers were present on ctg2. SSRs in hexameric form were the least distributed on the mitochondrial contigs, accounting for 3.23%, of which three hexamers were present on ctg1 and only one hexamer was present on ctg2. In addition, the pentameric form of the SSRs was found only on ctg1 (**Figure 2**). We identified 38 different repeating motifs among all SSRs in the *P. arundinacea* mitogenome, including mononucleotides (*n* = 10.5), dinucleotides (*n* = 8), trinucleotides (*n* = 3.4), tetranucleotides (*n* = 3), pentanucleotides (*n* = 1.3), and hexanucleotides (*n* = 1.3). A/T (*n* = 19) was the most common motif (15.32%), followed by AATG/ATTC (*n* = 16), 12.90%). While differing in repeat type and motif composition, these sequences shared a strikingly similar genomic arrangement. A total of 116 SSRs (93.55%) were located in the IGS, and a few others were distributed in intron and gene regions (**Supplementary Tables 5**, **S6**).

### Codon usage analysis of PCGs

**Figure 5:**
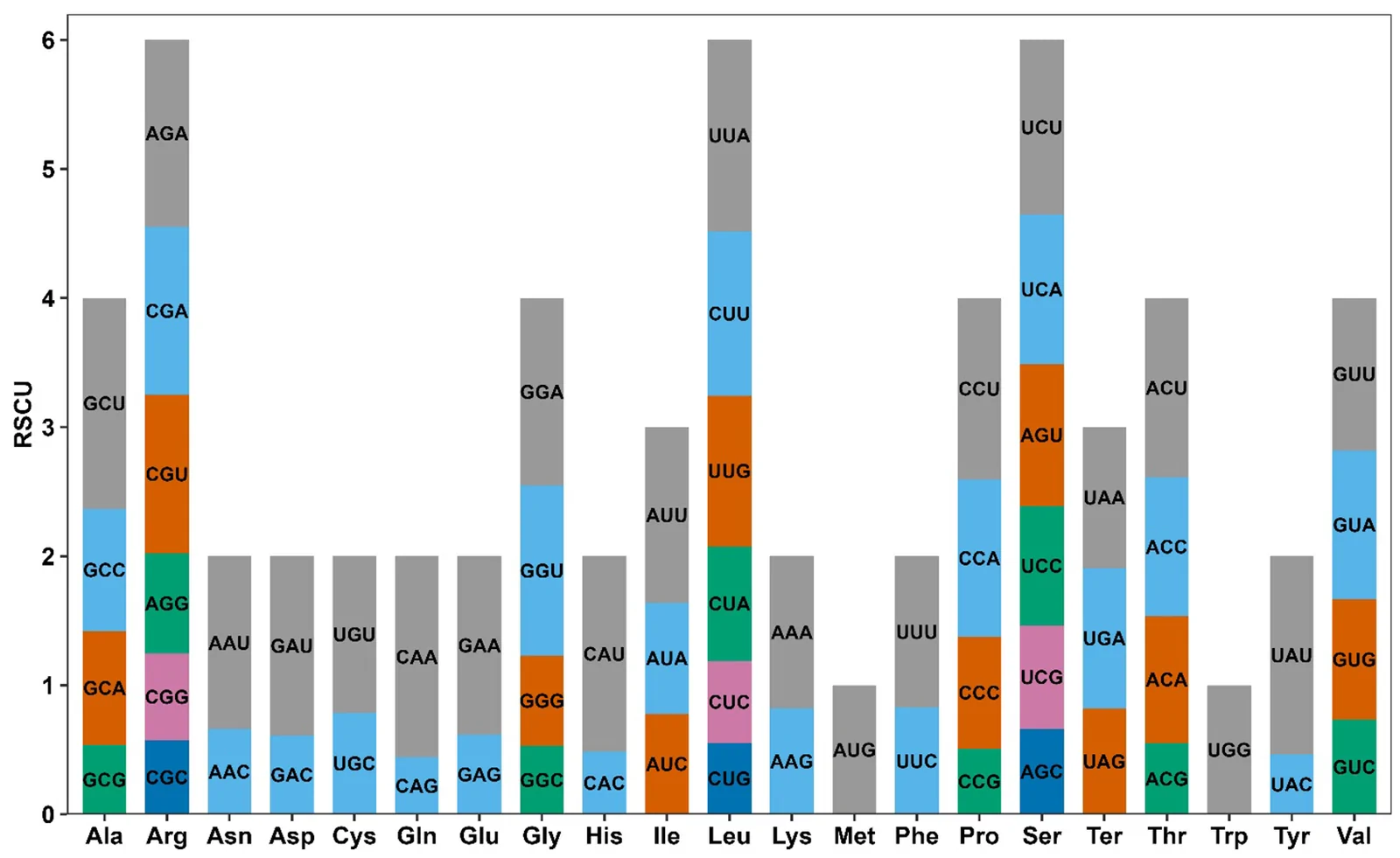
RSCU in PCGs of *Phalaris arundinacea* mitogenome. Codon families are on the abscissa.

RSCU is a metric of evolutionary pressures-including selection, mutation, and drift-acting on codon preference. In this study, we quantified the RSCU values for the *P. arundinacea* mitochondrial genome to assess its codon preference, where a value exceeding 1 denotes a positive bias toward a specific synonymous codon. In total, 37 unique PCGs in the *P. arundinacea* mitogenome are encoded by 9,779 codons. (**Supplementary Table 9**). The usage frequencies of the amino acids Leu, Ser, and Ile were the highest at 10.84%, 8.89%, and 7.90%, respectively. The frequency of stop codons was the lowest, at only 0.34%. Codon usage analysis confirmed the employment of all 64 codons in the mitogenome, split nearly equally between those with a preferential usage bias (RSCU > 1) and those, conversely, used at below-average frequencies (**Figure 5**). For example, one alanine codon is encoded by four codons. Codon preference levels varied in the mitogenome of *P. arundinacea*. Specifically, codon GCT exhibited a high usage preference, with an RSCU value of 1.63.

### RNA editing sites prediction

**Figure 6:**
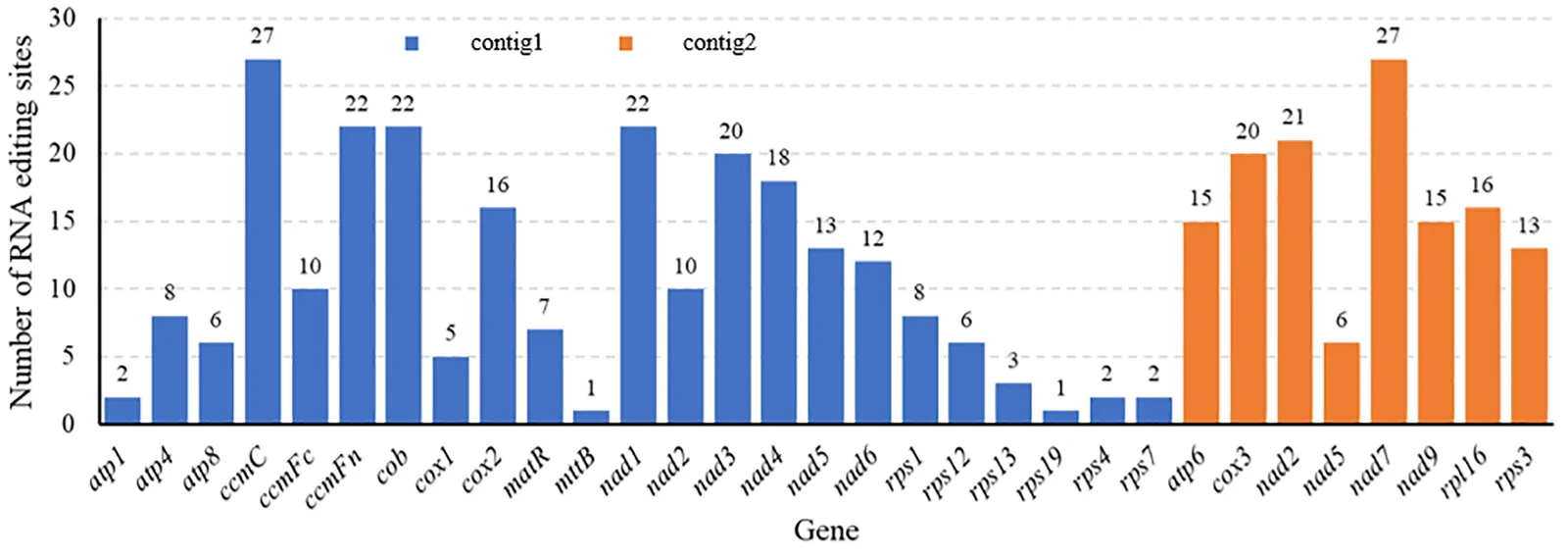
RNA editing sites in the *Phalaris arundinacea* mitochondrial PCGs.

**Figure 7:**
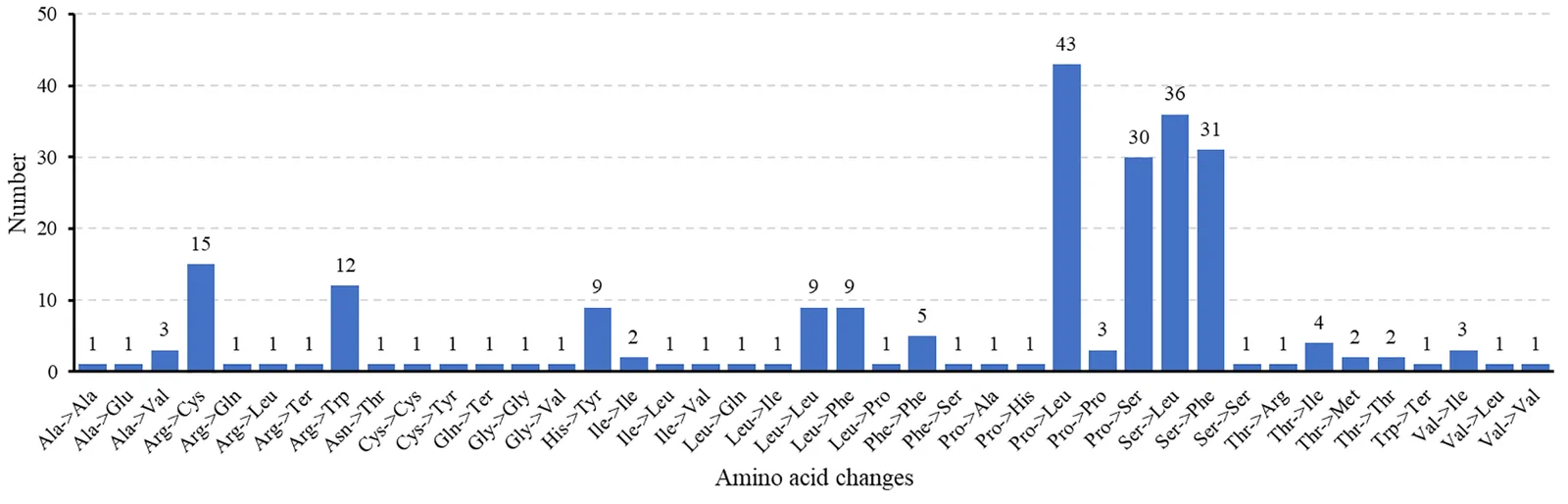
RNA editing-induced amino acid substitutions on *P. arundinacea* ctg1.

**Figure 8:**
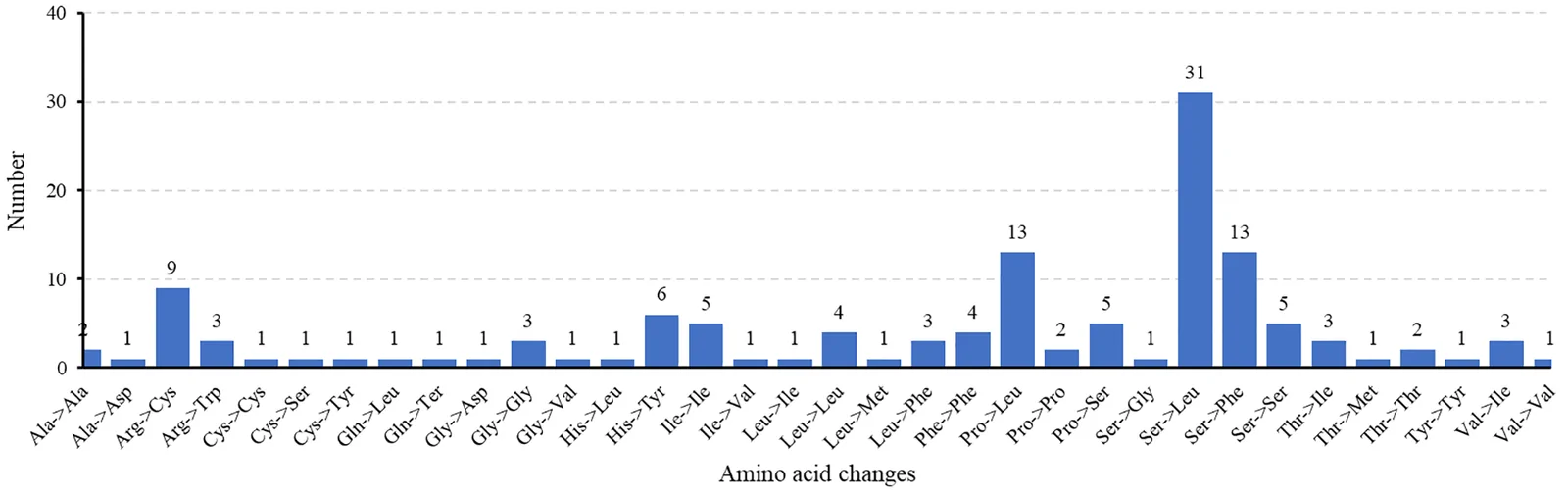
RNA editing-induced amino acid substitutions on *P. arundinacea* ctg2.

RNA editing is a widespread and frequent post-transcriptional process in plant mitochondria. We predicted and analyzed the RNA editing sites of shared PCGs in the mitochondrial genome of *P. arundinacea* and identified 376 valid RNA editing sites located in 31 PCGs. Correspondingly, 23 PCGs were distributed on ctg1 and eight PCGs were distributed on ctg2 (**Figure 6**). Among these, *ccmC* and *nad7* had the most RNA editing sites (located in ctg1 and ctg2, respectively), both with 27 sites, whereas *mttB* and *rps19* had only one RNA editing site each, both located on ctg1. Eleven types of changes were identified among the 376 RNA editing sites, mainly C-to-T changes (*n* = 333, 88.56%). Additionally, except for G-to-A changes (*n* = 15, 3.99%) and C-to-A changes (*n* = 8, 2.13%), the frequencies of the remaining editing checkpoint changes were all less than 4%. The positional statistics of the RNA editing sites revealed that most of these checkpoints occurred at the second position of the triplet codon (*n* = 200, 53.19%), followed by the first position (*n* = 124, 32.98%) (**Supplementary Table 10**). A total of 49 amino acid variants were found during RNA editing, and 41 amino acid variants appeared on ctg1 (**Figure 7**), including 10 synonymous substitutions and 3 stop codon changes. The most frequent amino acid substitution involved the conversion of proline to leucine (*n* = 43), followed by the conversion of serine to leucine (*n* = 36). In ctg1, the main amino acid variants were proline, leucine, serine, and phenylalanine (57.61%) residues. Although 34 amino acid variants were found on ctg2 (**Figure 8**), the most frequent substitution observed was from serine to leucine (*n* = 31); the remaining amino acid variants were all fewer than 14. These results indicate that *P. arundinacea* mitochondrial genomic RNA editing occurs on a relatively small scale and remains generally stable.

### Chloroplast-derived mitogenomic sequences

**Figure 9:**
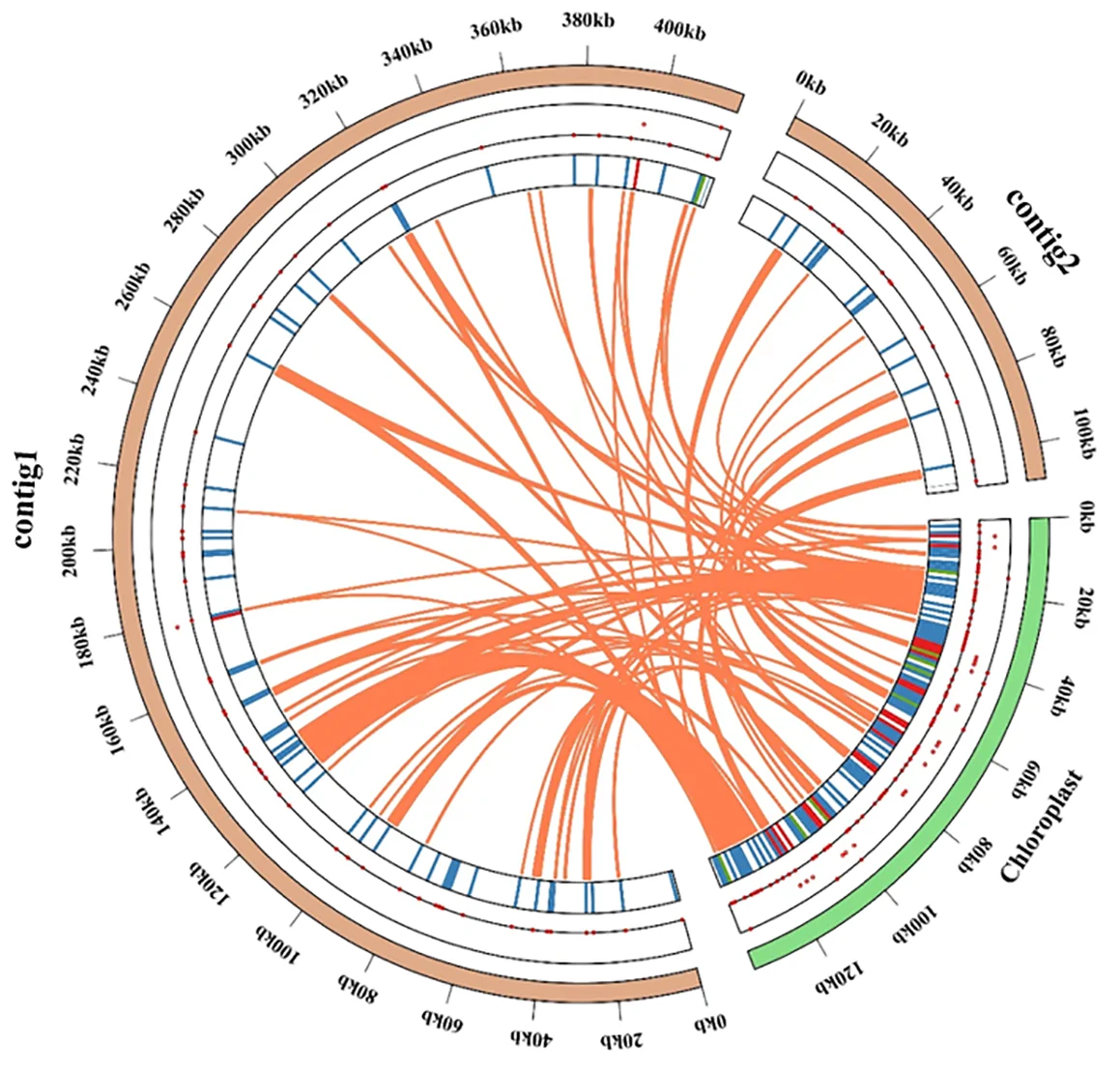
Inter-organellar gene transfer between the chloroplast and mitochondrial genomes. The visualization compares the chloroplast genome (light green segment) with the two mitochondrial contigs (purple segments). The internal heatmap density corresponds to the distribution of transferred sequences, with coral lines tracing the insertion routes of chloroplast-derived DNA into the mitogenome.

To elucidate the patterns of inter-organellar DNA transfer, homologous regions between the *P. arundinacea* mitochondrial and chloroplast genomes were compared and visualized. (**Figure 9**). We identified 104 fragments resulting from inter-organellar DNA transfer (**Supplementary Table 11**). These fragments mainly consist of gene spacers of protein-coding genes and contain small amounts of RNA and tRNA genes. The transferred sequences, spanning 55,866 bp (10.60% of the mitogenome), were overwhelmingly concentrated on ctg1 (86 out of 104 fragments). Notably, the two longest transfer fragments were located on ctg1. One fragment was transferred from the chloroplast (*rrn16, rps12, ndhB (intron), trnL-CAA, rrn23, trnT-CGU, trnA-UGC* (intron), *rps12* (intron), *ndhB, trnV-GAC, rps7, trnT-CGU* (intron), *trnA-UGC*) to the mitochondrial gene spacer (*trnA-UGC, trnL-CAA*). Another fragment was transferred from the chloroplast (*rrn23, trnL-CAA, trnA-UGC* (intron), *trnT-CGU, ndhB* (intron), *rrn16, rps12, trnV-GAC, ndhB, trnA-UGC, rps7, trnT-CGU* (intron), *rps12* (intron)) to the mitochondrial tRNA gene or its codon (*trnA-UGC* (intron), *trnI-GAU*, *trnL-CAA, trnA-UGC, trnV-GAC, trnI-GAU* (intron)). Both fragments were 10,974 bp in length and exhibited a high degree of sequence similarity (99.927%). In addition, the fragments with the highest transfer frequency were transferred from *rrn16* and *rrn23* in the chloroplasts to *rrn18* and *rrn26* in the mitochondria (12 and 10 times, respectively).

### Selection pressure analysis

**Figure 10:**
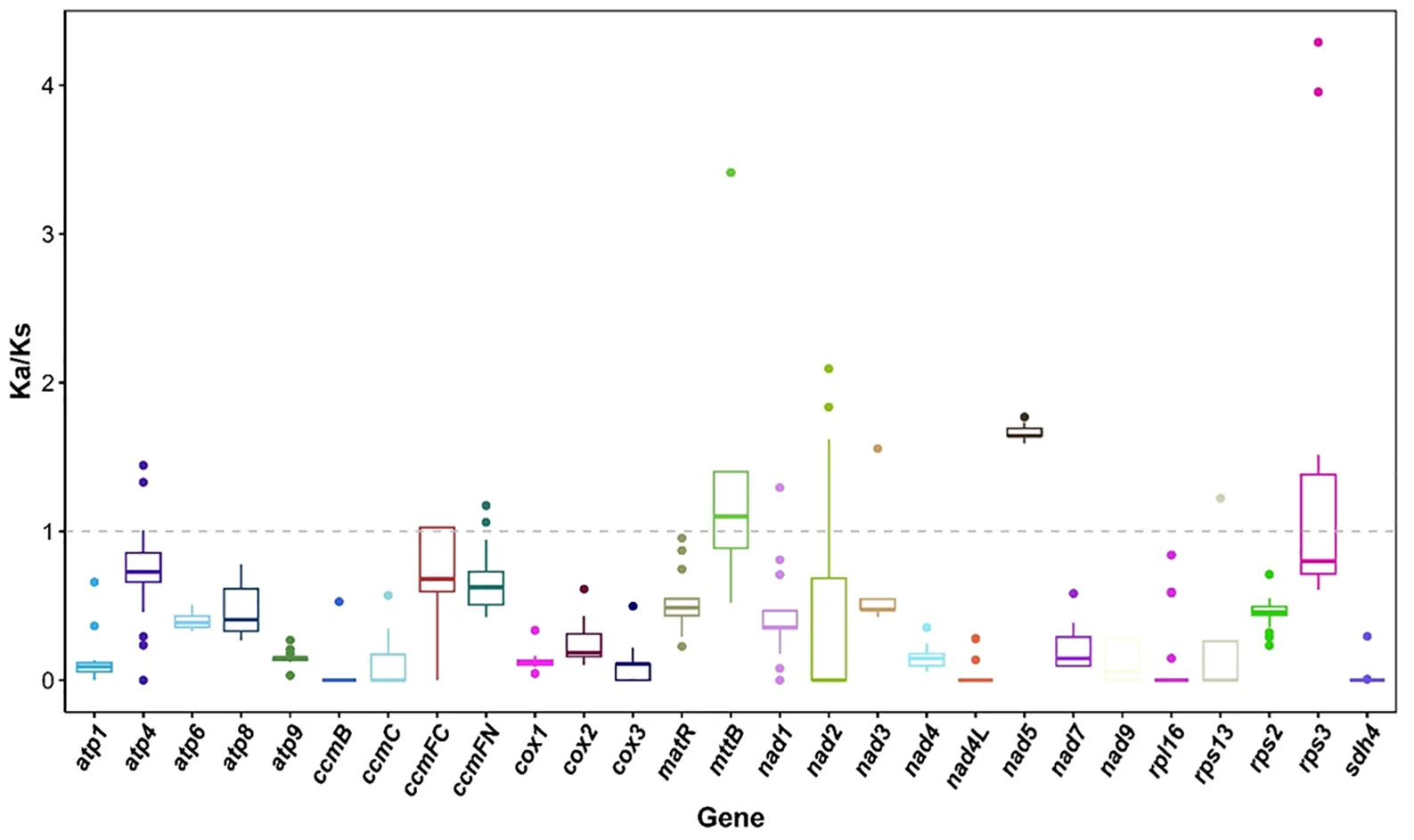
Boxplot of Ka/Ks values for 27 mitochondrial PCGs across 25 Poaceae species and *Phalaris arundinacea*.

Ka/Ks analysis was performed to evaluate the evolutionary selection pressures acting on 27 conserved PCGs across the mitochondrial genomes of 25 Poaceae species and *Phalaris arundinacea* (**Figure 10**). Most mitochondrial genes exhibited Ka/Ks < 1, suggesting that purifying selection represents the predominant evolutionary force maintaining mitochondrial gene conservation. However, several genes showed lineage-specific elevations in Ka/Ks ratios. In particular, *nad5* displayed Ka/Ks values greater than 1 across the sampled species, while *mttB* and *rps3* exhibited Ka/Ks values greater than 1 in specific lineages, especially within the Poa genus. These elevated Ka/Ks ratios may indicate potential adaptive divergence. Additionally, the Ka/Ks values of *ccmC*, *cox3*, and *rpl16* in *Aegilops cylindrica*, *Aegilops tauschii*, and *Agropyron cristatum* were 0, These values indicate an absence of detected nonsynonymous substitutions in the analyzed alignments, which may reflect strong functional conservation. However, such patterns can also result from limited sequence variation, short aligned regions, or insufficient numbers of informative sites.

Gene loss patterns among the 27 PCGs varied substantially (**Supplementary Table 12**). *Cynodon dactylon* lacked only *nad4L*, whereas *Eremochloa ophiuroides* and the three *Zea* species lost two genes (*ccmB* and *rpl16*). Extensive gene loss (six genes) occurred in *Aegilops tauschii*, *Elymus magellanicus*, *Fargesia qinlingensis*, and two *Poa* species, likely due to complete sequence conservation or the absence of polymorphic sites. These divergent Ka/Ks dynamics underscore the roles of both purifying and positive selection in shaping mitochondrial genome evolution across Poaceae, thereby providing critical insights into lineage-specific adaptive mechanisms.

### Nucleotide diversity

**Figure 11:**
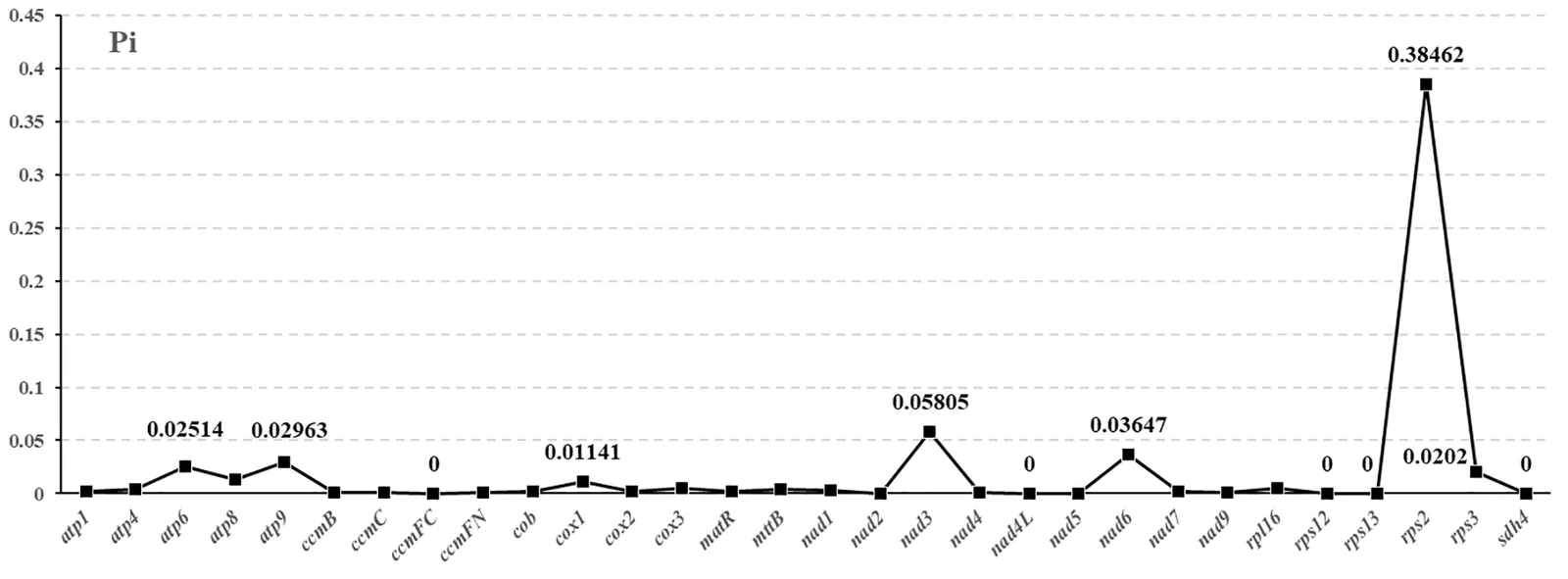
Pi of 30 PCGs in the mitochondrial genome of *Phalaris arundinacea*.

To further elucidate the genetic diversity characteristics of the mitochondrial genome of *Phalaris arundinacea*, we performed nucleotide diversity (Pi) analysis to assess sequence variation across the 30 PCGs (**Figure 11**; **Supplementary Table 13**). The Pi values across the PCGs spanned from 0 to 0.38462, with an overall mean of 0.0205, indicating low average nucleotide diversity. Among the PCGs, *rps2* showed the highest Pi value, followed in descending order by *nad3* and *nad6*, suggesting elevated functional specificity in mitochondrial mRNA translation and NADH dehydrogenase synthesis. In contrast, *ccmFC*, *nad4L*, *rps12*, *rps13*, and *sdh4* displayed Pi values of zero, indicating stringent evolutionary conservation. These conserved genes are critical for driving ATP synthesis and ensuring robust functionality of the electron transport chain.

### Phylogenetic analysis

**Figure 12:**
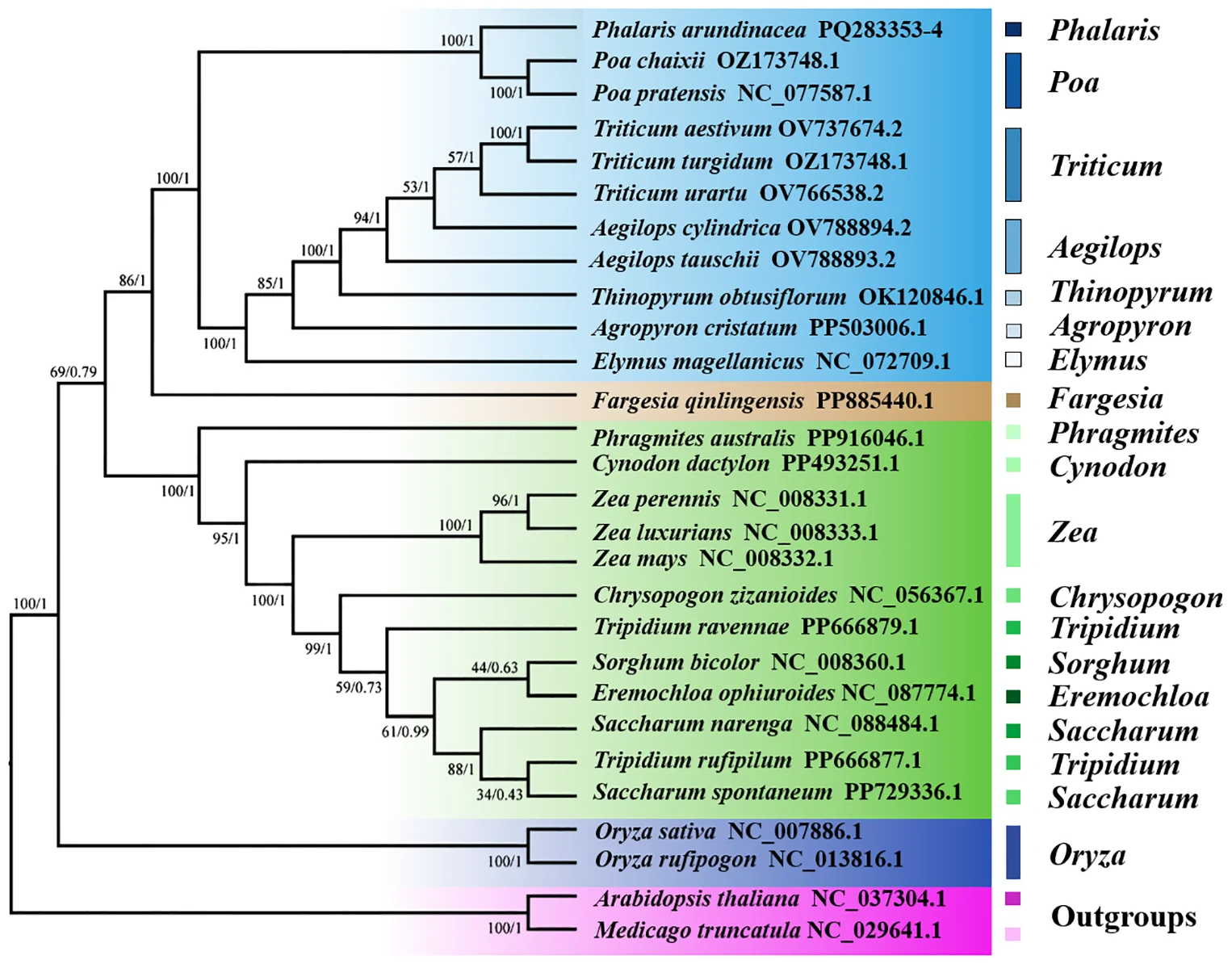
Phylogenetic position of *Phalaris arundinacea* among 28 species. Node labels show ML bootstrap values (left) and Bayesian posterior probabilities (right).

To investigate the evolutionary relationships of *P. arundinacea*, we selected 26 species of Poaceae, including *Phalaris arundinacea*, along with one species of Brassicaceae and one species of Fabaceae. We then integrated published mitochondrial genome data to construct a phylogenetic tree with Brassicaceae and Fabaceae species used as outgroups (**Figure 12**). The analysis revealed that the 26 species could be divided into three groups. The first group comprises *Fargesia, P. arundinacea, Poa, Triticum, Aegilops, Thinopyrum, Agropyron, Elymus, Phragmites, Cynodon, Zea, Chrysopogon, Tripsacum, Sorghum, Eremochloa*, and *Saccharum*. the second group comprises the genus *Oryza*, and the third group comprises the genera *Arabidopsis* and *Medicago*. Among the 25 nodes of the phylogenetic tree, the support rates of 15 nodes exceeded 90%, and 11 reached 100%. This tree clearly distinguished Poaceae plants from the outgroups (Fabaceae and Brassicaceae plants) and differentiated plants of different genera. *P. arundinacea* was identified within the first group. It showed a relatively close relationship with the genus *Poa* and was more closely related to the genera *Triticum, Aegilops, Thinopyrum, Agropyron*, and *Elymus* than to other genera. These findings contribute to resolving Poaceae phylogeny using mitochondrial genomes and provide a framework for investigating evolutionary relationships within the family.

### Synteny analysis

**Figure 13:**
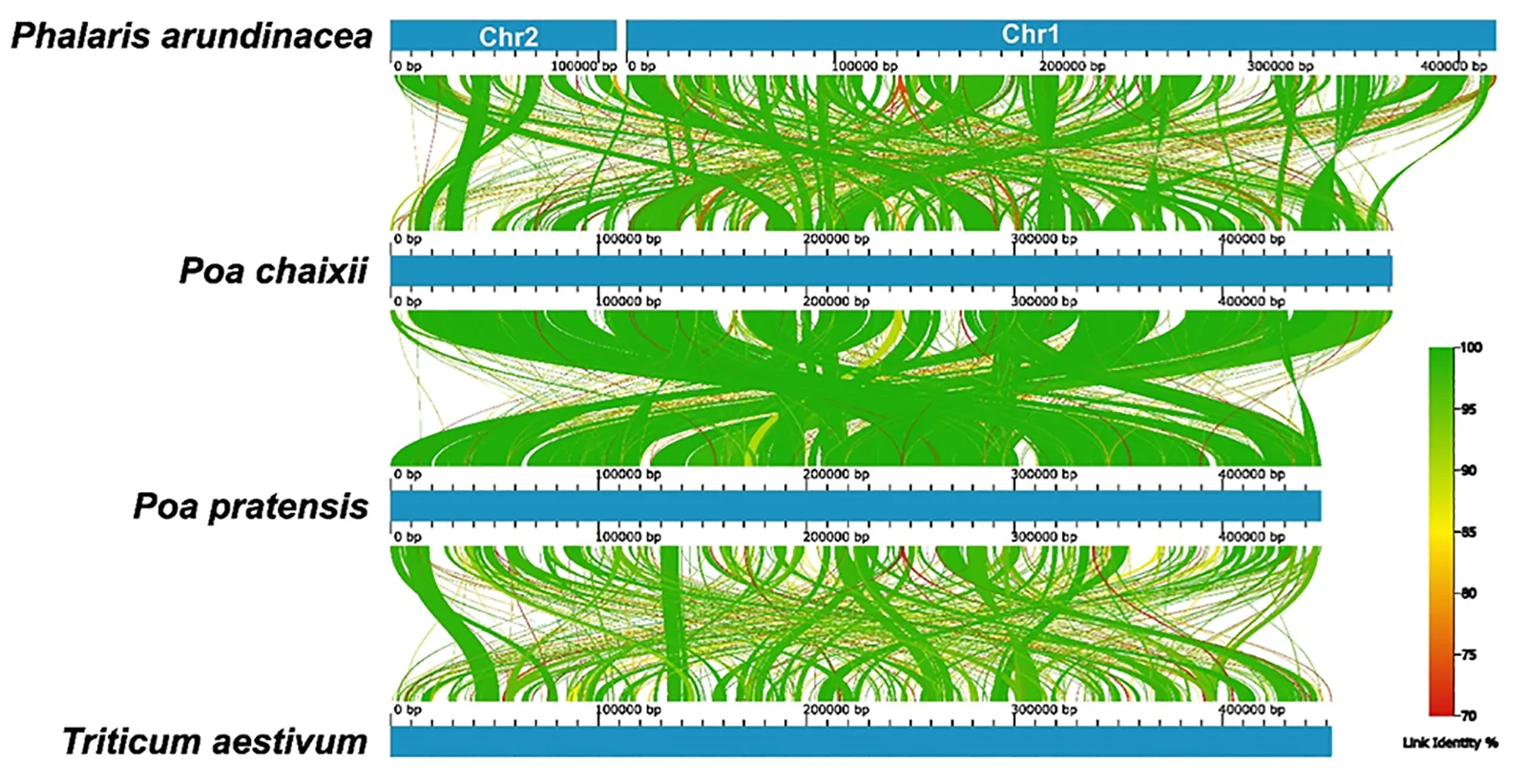
Comparative synteny analysis among *Phalaris arundinacea*, *Poa chaixii*, *Poa pratensis*, and *Triticum aestivum*. Sequence identity of syntenic blocks indicated by a green-to-red gradient (high to low).

Based on the phylogenetic tree results, a comparative synteny analysis was performed between *P. arundinacea* and three closely related species: *Poa chaixii*, *Poa pratensis*, and *Triticum aestivum* (**Figure 13**). Pairwise comparisons revealed that 63.84% of syntenic blocks in the mitochondrial genomes of *P. arundinacea* and *Poa chaixii* were homologous. The highest sequence homology (93.33%) was observed between *Poa pratensis* and *Poa chaixii*, thus validating the accuracy of the synteny analysis (**Supplementary Table 14**).

## Discussion

### Structural characteristics of the mitogenome in *P. arundinacea*

Resolving the complex evolutionary dynamics of organellar genomes-both plastid and mitochondrial-remains a pivotal challenge in modern phylogenomics. Benefiting from iterative advancements in high-throughput sequencing technologies, extensive high-quality assemblies of nuclear and chloroplast genomes across Poaceae species have been resolved to date. However, the elucidation of Poaceae mitochondrial genomes remains comparatively limited, largely constrained by their complex repeat sequences and frequent structural rearrangements (Gualberto, 2014; Mower, 2020). Most plant research has focused on the chloroplast genome (Liu et al., 2021). Similarly, the chloroplast genome of *Phalaris* has been sequenced (Xiong et al., 2020), but the mitochondrial sequencing of this species has not yet been completed. Accumulating evidence from prior studies has established that plant mitochondrial genomes exhibit an extraordinarily complex genomic architecture. For most land plant taxa, the mitochondrial genome exists *in vivo* in a state of dynamic equilibrium between a full-length master circle and heterogeneous subgenomic circles (Kozik et al., 2019; Tao et al., 2025)- a conformational feature that is postulated to facilitate DNA replication and damage repair or, alternatively, serve as a selective checkpoint during gametogenesis. This study assembled the mitochondrial genome of *P. arundinacea* into two circular structure of 526,717 bp with a GC content of 44.46%, thereby establishing a reference genetic resource for the species. Additionally, the mitochondrial genome of barley (GenBank accession numbers PQ283353 and PQ283354) was 525,599 bp in total length and 44.2% GC (Hisano, 2016), which is very close to that of *Phalaris arundinacea*. Comparative analysis revealed that the *P. arundinacea* mitogenome is larger than that of many sequenced Poaceae species (**Supplementary Table 1**). This structural feature indicates fewer introns and limited sequence transfers in the *P. arundinacea* mitogenome, which may reflect a more conserved evolutionary trajectory within the Poaceae family. The mitogenome of *P. arundinacea* exhibits a double-ring architecture, as also observed in *Setaria italica* (Zhang, 2024) and *Prunella vulgaris* (Sun et al., 2023). During mitochondrial assembly, the appearance of double-ring structures is caused by the active recombination of repeating sequences (Zhang, 2024). The mitochondrial genome of Ice Grass was reported to contain 35 protein-coding genes (Ou et al., 2024), while 36 were identified in that of *Ventilago leiocarpa* (Guo et al., 2024). By contrast, our annotation of the *P. arundinacea* mitogenome identified 16 core and 21 variable protein-coding genes. The observed disparity could be attributed to evolutionary DNA transfer events among the plastid, nuclear, and mitochondrial compartments. Mitochondria are the dominant source of intracellular reactive oxygen species (ROS), a major driver of DNA damage and mutagenesis, making the mitochondrial genome particularly vulnerable to spontaneous and recurrent mutations (Pakendorf and Stoneking, 2005). The endosymbiotic transfer of core mitochondrial genes to the nuclear genome-which benefits from far more stringent genome surveillance, superior protection from mutagenic insults, and more sophisticated DNA damage repair pathways-is widely recognized as a key adaptive evolutionary benefit in eukaryotes (Allen, 2015). Notably, the mitochondrial genome of *P. arundinacea* harbors a reduced complement of annotated genes relative to some other Poaceae lineages. This observation raises the possibility that *P. arundinacea* may have experienced a relatively extensive process of mitochondrial-to-nuclear gene transfer during the course of Poaceae diversification, although alternative explanations - such as gene loss or annotation limitations - cannot be ruled out.

### Diversity and variation in the mitogenome of *P. arundinacea*

Our structural dissection of the *P. arundinacea* mitogenome unveiled a substantial proliferation of repetitive elements, spanning simple, tandem, and dispersed iterations. These repetitive sequences are known to drive mitochondrial genome recombination across various scales and at differential abundance (Alverson et al., 2011). Moreover, these repeats can serve as effective molecular markers for genetic analysis owing to their specific sequence signatures (Bi et al., 2016; Ma, 2022). For example, SSRs are often used as molecular markers because of their unique genetic characteristics and lack of environmental influence (Song et al., 2015). In recent years, these markers have been widely used to analyze and identify herbs (Chen, 2022). Mitochondrial genome size shows a significant positive correlation with the count of repeat sequences exceeding 500 bp in length (Allen et al., 2007; Alverson et al., 2024). Through an in-depth investigation of the mitochondrial genome of *Phalaris arundinacea*, we accurately identified 318 repeats. With a total length of 526,717 bp, the mitogenome contained repeat sequences accounting for over 20% of its sequence, highlighting their substantial contribution to its architecture. Illustrating this correlation, the notably larger mitochondrial genome of zucchini (982,833 bp) contains a high density of repeats, accounting for up to 38% of its sequence (Allen et al., 2007). By analyzing the repeats in the mitochondrial genomes of these different species, we further confirmed the close relationship between repeat sequence size and mitochondrial genome size, providing a solid theoretical basis and data support for subsequent research in related fields. The SSR markers identified in this study not only enrich the fundamental genetic dataset for *Phalaris arundinacea* but also provide a robust and directly applicable molecular toolset for three key research and breeding applications: evaluating genetic diversity across wild *P. arundinacea* populations, reconstructing its invasion pathways, and enabling marker-assisted selection in the breeding of elite forage cultivars.

RNA editing constitutes a hallmark feature of plant mitochondrial genomes and can be regarded as template-based post-transcriptional regulation involving single-base switching mode (Zhang, 2024). It can also promote changes in the mitochondrial gene expression patterns (Handa, 2003). A total of 376 RNA editing sites were identified in the *P. arundinacea* mitogenome, a number comparatively fewer than those reported for *Setaria italica* (*n* = 417) (Zhang, 2024) and *Poa pratensis* (*n* = 457) (Ai et al., 2024). In plants, mitochondrial RNA editing depends on the extensively expanded pentatricopeptide repeat (PPR) protein family, whose members bind and modify these target sites with single-nucleotide precision. The primary evolutionarily conserved role of this process is to correct deleterious mutations in the mitochondrial genome, thereby restoring functionally critical conserved amino acid residues in the encoded proteins and maintaining the integrity of mitochondrial respiratory chain function (Steinhauser et al., 1999). The predominant RNA editing event involved C-to-U conversions, primarily at the first and second codon positions. This pattern aligns with the canonical profile reported for plant mitochondrial genomes (Sloan et al., 2010; Small et al., 2020; Ou et al., 2024). RNA editing mainly affected functional genes related to energy metabolism, cytochrome c synthesis, mitochondrial gene expression regulation, and ribosome composition in the mitochondrial genome of *Phalaris arundinacea*, involving a total of 29 protein-coding genes. Relative to the mitochondrial genome of *Agropyron cristatum* (Ou et al., 2024), we detected RNA editing events across a larger set of functional PCGs in the *P. arundinacea* mitogenome. This discrepancy may be attributed to the adaptive evolution of *P. arundinacea* to its native habitats: floodplains, marshes and other wetland environments that are frequently subject to acute hypoxia and submergence stress. The highly efficient RNA editing system serves as a critical post-transcriptional regulatory mechanism that maintains the correct coding sequence of respiratory chain-related genes, thereby ensuring the functional assembly and integrity of the mitochondrial respiratory chain under adverse environmental conditions.

Contrasting with animal mitochondrial genomes, plant mitochondrial genomes display a reduced mutation rate (Darracq et al., 2011) and also incorporate DNA fragments from other cellular compartments, such as the chloroplast (Law et al., 2022). Chloroplast gene fragments have been found in the mitochondrial genomes of many species; however, their transfer varies widely among species (Cheng et al., 2021). In bluegrass, 31 chloroplast-derived segments were transferred to the mitochondrial genome, with 14 of these being complete fragments (Ai et al., 2024); Similarly, in *Suaeda salsa*, 32 mitochondrial genome segments were identified as having originated from the chloroplast genome, encompassing eight annotated genes (Cheng et al., 2021). A substantial amount of chloroplast DNA was identified in the mitogenome, comprising 104 fragments with a cumulative length of 55,866 bp, which accounts for about 10% of the mitochondrial genome sequence, indicating frequent fragment transfer between mitochondria and chloroplasts. However, gene transfers can have different outcomes. Occasionally, the function of the transferred gene remains intact. In other instances, gene structure undergoes modifications. Moreover, there are various types of gene transfer, such as those occurring from entire genes to gene spacer regions.

### Evolutionary classification of *P. arundinacea* based on the mitogenome

Codon degeneracy allows multiple nucleotide sequences to encode the same amino acid, while the choice among synonymous codons is often biased. Several factors-gene expression, gene length, tRNA pool, and codon position-collectively contribute to shaping the distinct codon usage bias observed in different organisms (Sun et al., 2023). Codon usage preferences represent trends in base composition and natural selection in the genome, providing a theoretical basis for species-specific evolution. In the mitochondrial genome of *Phalaris arundinacea*, the codon with the highest usage preference in the mitochondrial genome of *P. arundinacea* was GCT, followed by CAA, TAT, CAT, and TTA, similar to *Agropyron cristatum* (Ou et al., 2024) and *Quercus acutissima* (Liu et al., 2022). Interestingly, codon shifts toward G/C bases were more frequent than those toward A/T. This bias toward higher GC content could contribute to enhanced gene stability. The biological significance of this strong GC preference is not restricted solely to enhancing gene stability. In plant mitochondria, this characteristic is predominantly driven by GC-biased gene conversion (gBGC) (Liu et al., 2020). This is a molecular mechanism tightly coupled to the homologous recombination repair of DNA double-strand breaks (DSBs): when ROS-induced mitochondrial DNA DSBs are repaired via homologous recombination, the mismatch repair system exhibits a consistent insertion preference for G/C bases (Lesecque et al., 2013). The long-term evolutionary accumulation of this effect ultimately shapes the high GC content of the mitochondrial genome and the GC bias in codon usage.

To delineate the selective pressures acting upon the mitochondrial coding sequences, Ka/Ks metrics were calculated, serving as robust indicators of evolutionary constraint versus adaptive divergence. By quantifying the divergence between Ka and Ks substitution rates, this approach enables systematic evaluation of sequence conservation and selection pressure patterns across homologous genes in divergent species (Wang et al., 2010). Evolutionary selection pressure analyses of 27 conserved genes in Poaceae (including *Phalaris arundinacea* and 25 related species) revealed that over 70% of the genes were under strong purifying selection (Ka/Ks < 1), whereas the *nad5* gene exhibited positive selection signals (Ka/Ks > 1) across all species. The *nad5* gene encodes the core transmembrane subunit of mitochondrial respiratory chain Complex I (NADH dehydrogenase), which is responsible for proton translocation across the inner mitochondrial membrane (Tsai et al., 2022). The vast majority of plant respiratory chain genes are under intense purifying selection (Ka/Ks < 1) to maintain essential cellular functions required for organismal survival (Gualberto and Newton, 2017). In *P. arundinacea*, however, *nad5* exhibited Ka/Ks values greater than 1 across the analyzed species, suggesting a possible signal of adaptive divergence. However, elevated Ka/Ks ratios should be interpreted with caution, as they may also arise from stochastic variation, low synonymous substitution rates, alignment uncertainty, or differences in taxonomic sampling. Therefore, although *nad5* represents a potential candidate gene associated with lineage-specific mitochondrial evolution, additional population-level analyses and functional investigations are required to confirm whether adaptive selection contributed to its diversification. The functional significance of the observed nad5 variation remains unclear and requires further investigation using broader taxonomic sampling and functional validation. This finding provides a key target for future research on energy metabolism in *Poaceae* plants. Furthermore, synonymous substitutions predominate in plant mitochondrial genomes, and genetic variations in critical functional genes are strictly constrained by purifying selection, indicating their functional irreplaceability (Ou et al., 2024). Ka/Ks ratio analysis enabled the precise identification of adaptive divergence patterns in orthologous PCGs across species, providing molecular evidence for deciphering lineage-specific evolutionary trajectories.

To validate the taxonomy of *Phalaris* plants using their mitochondrial genome, we constructed a phylogenetic tree containing 28 plant mitochondrial genomes. Earlier studies classified *Phalaris* within the oat-bluegrass complex of the subfamily Pooideae (Quintanar et al., 2007; Schneider et al., 2009; Saarela et al., 2015), In recent years, *Phalaris arundinacea* has been reported to possess multiple ploidy levels, although tetraploid dominates. As a result, some taxonomists have recognized two taxonomic categories of *Phalaris arundinacea*: subspecies and variants (Perdereau et al., 2017). Some scholars believe that *Ph*alaris exhibits aneuploidy, some forms of which may have evolved through diploidy. The genus lacks subordinate classification, and species relationships and subordinate divisions should be evaluated using with multidisciplinary information, such as morphology (Handa, 2003; Voshell and Hilu, 2014)Phylogenetic analyses revealed that *Phalaris* is relatively closely related to *Poa* and forms a separate branch, which is consistent with previous results. Furthermore, comparative synteny analysis provided structural genomic evidence supporting this phylogenetic relationship. The results revealed that 63.84% of the syntenic blocks between the mitochondrial genomes of *P. arundinacea* and *Poa chaixii* were homologous. This finding confirms a hallmark evolutionary characteristic of plant mitochondrial genomes: an extremely low nucleotide substitution rate coupled with an exceptionally high structural rearrangement rate (Palmer and Herbon, 1988). Frequent structural rearrangements also act as the underlying cytological driver for the occurrence of polyploidy and the complex infrageneric classification within the *Phalaris* genus. The highest sequence homology (93.33%) was observed between *Poa pratensis* and *Poa chaixii* within the genus *Poa*, validating the accuracy of the current synteny analysis. These findings support the close phylogenetic relationship between *P. arundinacea* and species of the genus Poa. The mitochondrial genome data generated in this study provide a valuable reference and a foundation for future phylogenetic studies within Phalaris that incorporate additional chloroplast, nuclear, or mitochondrial genome data from a broader representation of Phalaris species.

## Conclusions

This study presents the first assembly and structural characterization of the *P. arundinacea* mitochondrial genome, a circular molecule of 526,717 bp harboring 72 genes (37 PCGs, 27 tRNAs, and 8 rRNAs). Subsequently, we investigated the dynamic aspects of the *P. arundinacea* mitogenome by analyzing repeat sequences, RNA editing sites, and inter-organellar gene transfer. Furthermore, through analyses of codon usage bias, nucleotide diversity, and phylogenetics, we assessed its evolutionary trajectory and phylogenetic placement within the Poaceae. Collectively, this work provides foundational insights into the genetic evolution of *P. arundinacea* and addresses a key gap in its genomic resources. It also provides a solid theoretical foundation for subsequent genetic breeding exploration and offers valuable scientific guidelines for the mining and application of the valuable genetic characteristics contained in *P. arundinacea*.

## Data Availability

The datasets presented in this study can be found in online repositories. The names of the repository/repositories and accession number(s) can be found in the article/**Supplementary Material**.

## Supplementary material

Supplementary Table 1 GenBank accession numbers of plant mitogenomes sampled in this study.

Supplementary Table 2 Gene composition in the *Phalaris arundinacea* mitogenome.

Supplementary Table 3 The start codon and stop codon information of *Phalaris arundinacea* mitochondrial gene.

Supplementary Table 4 Size of introns and exons in split genes in *Phalaris arundinacea* mitogenome.

Supplementary Table 5 Microsatellite repeats in *Phalaris arundinacea* mitogenome.

Supplementary Table 6 Distribution of SSRs in *Phalaris aru*ndinacea mitogenome.

Supplementary Table 7 Dispersed repeats in *Phalaris arundinacea* mitogenome.

Supplementary Table 8 Distribution of tandem repeats in *Phalaris arundinacea* mitogenome.

Supplementary Table 9 Codon counts in the Phalaris arundinacea mitochondrial PCGs.

Supplementary Table 10 RNA editing sites in the mitogenome of *Phalaris arundinacea*.

Supplementary Table 11 Fragments transferred from chloroplast to mitochondria in *Phalaris arundinacea*.

Supplementary Table 12 The pairwise Ka/Ks values among the 25 Poaceae species.

Supplementary Table 13 The genetic diversity characteristics of the mitochondrial genome in *Phalaris arundinacea*.

Supplementary Table 14 The collinearity proportion of the genomes of four species.
